# Echocardiographic assessment after left bundle branch area pacing: Impact on the tricuspid valve apparatus, clinical value, and perspectives

**DOI:** 10.1016/j.hroo.2025.11.027

**Published:** 2025-12-10

**Authors:** Taïna Barre, Romain Pierrard, Anne Suzat, Naguib Benguella, Rayan Mohammed, Geoffrey Bayard, Kasra Azarnoush, Nathalie Grand, Marion Murat, Marouane Bouhkris, Karim Benali, Antoine Da Costa

**Affiliations:** Department of Cardiology, Jean Monnet University, Saint-Etienne, France

**Keywords:** Tricuspid regurgitation, Cardiovascular implantable electronic devices, Echocardiography, 3D-Echocardiography, Defibrillator, Pacemaker, Tricuspid valve, Left bundle branch area pacing

## Abstract

**Background:**

The impact of left bundle branch area pacing (LBBAP) on the tricuspid valve apparatus (TVA) have not yet been entirely investigated.

**Objective:**

This prospective study aimed to: (1) compare the impact of LBBAP and right ventricular apical pacing (RVAP) leads on TVA; (2) evaluate the incidence of new-onset lead-induced tricuspid regurgitation (LITR) or worsening of preexisting TR (WTR); and (3) assess the early impact of pacing type on left ventricular (LV) function (4) detect early complications.

**Methods:**

This observational study included consecutive patients undergoing either RVAP or LBBAP lead implantation. 3-dimensional transthoracic echocardiography (3D-TTE) was performed before and the day after cardiac implantable electronic devices (CIED) implantation.

**Results:**

A total of 115 patients were enrolled: 45 received LBBAP and 70 patients received an RVAP lead. Lead–leaflet impingement was observed in 49 of 115 patients (42.6%): 28 of 70 (40%) in the RVAP group and 21 of 45 (46.7%) in the LBBAP group (ns). In the LBBAP group, leads crossed the TV more frequently in the anterior area (19/45; 42%), whereas RVAP leads crossed more often in the posteroseptal area (50/70; 71.5%), (*P* = .025). The incidence of new-onset LITR or WTR was low (8%, 5/66) but represented 19% (5/26) of patients with leaflet impingement (*P* = .016). LBBAP was associated with an immediate LV function beneficial effect. Post-implantation TTE revealed complications including 3 CIED-related Takotsubo syndrome.

**Conclusion:**

This study found that LBBAP leads cross the tricuspid valve more frequently in the anterior area, whereas RVAP leads tend to cross in a posteroseptal position. Early worsening of TR after CIED implantation was uncommon.

**Key Findings**

▪ Left bundle branch area pacing (LBBAP) leads more frequently crossed the tricuspid valve at an anterior site, whereas right ventricular apical pacing (RVAP) leads tended to cross in a more postero-septal position.
▪ The early incidence of new-onset lead-induced tricuspid regurgitation (LITR) or worsening of preexisting TR (WTR) appeared to be low (4.3%, 5/115), but represented 10.2% (5/49) of patients with leaflet or commissural impingement identified.
▪ An immediate beneficial effect of LBBAP on indexed left ventricular stroke volume (SVi), post-implantation global longitudinal strain (ΔGLS), and time-to-peak strain dispersion was evidenced.
▪ Early systematic transthoracic echocardiography to detect complications is essential after device implantation, particularly for LBBAP.

## Introduction

Cardiac implantable electronic devices (CIEDs) such as permanent pacemakers (PPM), implantable cardioverter defibrillators (ICDs), and cardiac resynchronization therapy (CRT) systems are the cornerstone of the management of heart failure, arrhythmias and conduction disturbances.^1^ It is now well-documented that CIEDs are associated with tricuspid regurgitation (TR).^1^, ^2^, ^3^, ^4^ In a recent meta-analysis, the incidence of TR worsening by one grade was reported as 25%, and by 2 grades as 9.4%.^2^ Moreover, CIED-related TR has been shown to increase mortality by approximately 140%.^3^ TR can be classified as “CIED-associated”, when it results from coexisting cardiac comorbidities, mainly right ventricular (RV) dysfunction and/or dilation and right atrial dilation. Conversely, it is defined as “CIEp-related" when the cardiac device itself is the primary cause of regurgitation. Mechanisms of CIED-related TR fall into 3 types.^1^ “Implantation-related TR” encompasses leaflet perforation, impingement, adhesion, perforation, laceration or subvalvular entanglement. “Device-related TR” includes complications such as endocarditis, fibrosis, and leaflet avulsion during transvenous lead extraction (TLE).^5^ “Pacing-related TR” is induced by dyssynchrony.^1^, ^2^, ^3^, ^4^, ^5^ Among these, impingement remains the most common mechanism of CIED-related TR, predominantly affecting the posterior and septal leaflets.^1^, ^2^, ^3^, ^4^, ^5^, ^6^ A comprehensive imaging protocol is required to accurately assess the interaction between the lead, the tricuspid valve (TV) leaflets, and their supporting apparatus. This evaluation primarily relies on 2- and 3-dimensional (3D) transthoracic echocardiography (TTE) with and without color Doppler,^7^ whereas 3D transesophageal echocardiography (TEE) is often necessary in severe TR evaluation.^8^ In patients with RV apical lead implantation, it is known that the lead most commonly crossed the TV centrally, impinging on the septal leaflet in 23% of cases and on the posterior leaflet in 20%. Impingement of the anterior leaflet was observed in fewer than 5% of cases.^6^ Traditional RV apical pacing (RVAP) has been associated with deterioration of both left ventricular (LV) diastolic and systolic function.^9^ This decline is frequently accompanied by an increased risk of heart failure-related hospitalization, atrial fibrillation, and overall morbidity. Recently, attention has shifted toward more physiological pacing strategies, initially with His bundle pacing and, more recently, left bundle branch area pacing (LBBAP). These approaches provide more physiological and synchronized ventricular activation, leading to improved preservation of LV ejection fraction (LVEF) and reduced mitral regurgitation.^10^ In addition, His bundle pacing has been reported to decrease cardiac implantable electronic device (CIED)-related TR in at least one study.^11^ To date, only a few studies have investigated the impact of lead positioning on the development of lead-induced TR but failed to demonstrate a difference.^12^^,^^13^ Only Li et al^13^ found that the distance between the LBBAP lead fixation site and the TV might influenced the incidence of LBBAP lead-related TR. Indeed, data specifically addressing the interaction between the TV apparatus (TVA) and LBBAP remain limited, as this pacing modality is relatively new.^13^

The aims of this prospective study were the following: (1) to compare the impact of LBBAP and RVAP leads on the TVA; (2) toevaluate the incidence of new-onset lead-induced TR (LITR) or worsening of preexisting TR (WTR); (3) to assess the early impact of pacing type on LV remodeling function and monitoring for complications; and (4) to detect early complications.

## Methods

### Study population

This prospective single-center observational study included consecutive patients undergoing CIED implantation at the University Hospital of Saint-Étienne between October 2024 and October 2025. This study was conducted in accordance with the Declaration of Helsinki on ethical principles for medical research involving human subjects and was approved by the institutional review board of Saint-Etienne University. All patients provided written informed consent prior to participation.

### Inclusion criteria

Consecutive patients were prospectively enrolled if they received a CIED delivered by LBBAP or RV septal pacing.

### Exclusion criteria

Patients with preexisting TR ≥grade 2, significant valvular disease requiring surgery, poor echogenicity, history of surgical or percutaneous TV repair, or a previous CIED were excluded.

### Data collection

Baseline demographic characteristics, medical history, and type of cardiomyopathy were collected at admission. Standard 12-lead electrocardiograms (ECGs) were performed before and after implantation, prior to discharge.

### Echocardiographic assessment

A complete 3D-TTE was performed in the 24 hours preceding CIED implantation using the Vivid E95 ultrasound system (General Electric Healthcare, Chicago, Illinois, USA) with the 4-VTD 3-dimensional probe. LV volumes including indexed LV end-diastolic volume (LVEDVi), indexed LV end-systolic volume (LVESVi), and indexed left stroke volume (SVi) and LVEF, longitudinal strain parameters, including global longitudinal strain (GLS) and time-to-peak strain dispersion (tPSD), standard diastolic parameters, right and left atrial dimensions and longitudinal strain, gradation of aortic, mitral and TRs, RV systolic function parameters were assessed.

A second complete 3D-TTE was performed in the 24 hours following CIED implantation, using the previously described dataset. Lead TV crossing site and lead–leaflet interactions were meticulously assessed with 3D TV acquisitions, when possible with the multibeam mode, long- and short-axis 2D apical, parasternal, and, as far as possible, sub-xiphoid acquisitions. New-onset or worsening TR was carefully assessed. Grading of TR was identified on the basis of the 2017 American Society of Echocardiography recommendations.^5^ Significant TR was defined as at least a 1-grade increase to moderate or severe TR with at least a 1-grade increase after implantation.^5^, ^6^, ^7^, ^8^ All TTE were performed by 2 echocardiography specialists and reviewed by an expert team for 3D lead localization when discrepancies occurred. Lead–leaflet impingement was defined as the lead being visibly displaced by the leaflet during systole and/or when the lead clearly restricted systolic leaflet motion, regardless of whether TR was present. In rare cases with short commissures, impingement at the commissures was diagnosed only when both criteria were met. Anterior TV crossing was defined as anterior leaflet impingement, anteroposterior commissural crossing, or mid leaflet crossing. Postero-septal TV crossing included all other crossing sites, with or without impingement.

### Follow-up

A new complete TTE follow-up was performed at 3 or 6 months after implantation if possible. When the first post-implantation 3D-TTE did not allow us to localize the TV crossing site, new 3D acquisitions were made. TEE was performed to refine lead localization and assess potential lead-related TR mechanisms. All patients provided new informed consent before this additional invasive procedure which was performed exclusively for study purposes. Examinations were performed by a cardiac imaging specialist. Compared with TTE, TEE—particularly transgastric views—allowed superior accuracy for lead localization without systematic reliance on 3D acquisitions.

### Statistical analysis

We evaluated differences between patients implanted with a LBBAP device and those implanted with either an apical lead or a CRT device (RVAP group). All patients were electronically paced by their device. LV volumes (LVEDV, LVESV, and stroke volume), indexed to body size, and LVEF were collected. Global longitudinal strain and time-to-peak dispersion were also assessed. For each parameter, a Δ (day1–day0) was calculated. Descriptive statistics (means ± standard deviation, medians with ranges and quartiles) were generated for each group and for the overall cohort. Group comparisons were performed using ANOVA when assumptions of normality were met, or the Kruskal-Wallis test otherwise. When the overall test was significant, post hoc pairwise analyses were carried out with Dunn’s test (non-parametric). *P*-values were adjusted for multiple comparisons using the Bonferroni correction, and values <.05 were considered statistically significant.

## Results

### Population characteristics

The study enrolled 115 patients: 45 received a LBBAP device, 20 received an ICD (including 7 with CRT), and 50 underwent RVAP (of whom 7 also had CRT). Baseline characteristics of the study population are presented in Table 1, and baseline electrocardiographic data are summarized in Table 2. All patients underwent pre- and post-implantation 3D-TTE, but only 66 of 115 (57.4%) had follow-up TTE between 3 and 6 months after implantation. Seven patients (6.1%) required and underwent a post-implantation TEE to precise the lead localization.

**Table 1:** Baseline characteristics of the study population

Characteristics of the study population	Total (n = 115)	LBBAP (n = 45)	RVAP (n = 50)	ICD (n = 20)	*P*-value
Men	75 (65%)	30 (67%)	28 (56%)	17 (85%)	.068
Age, y (±SD)	76 **±** 9.8	80 **±** 10.2	80 **±** 7.7	69 **±** 9.2	<.001
High blood pressure	73 (63%)	32 (71%)	29 (58%)	12 (60%)	.390
Diabetes	23 (20%)	12 (27%)	5 (10%)	6 (30%)	.061
Overweight	22 (19%)	8 (18%)	9 (18%)	5 (25%)	.763
Mitral or aortic valve surgery	8 (7%)	5 (11%)	2 (4%)	1 (5%)	.391
Heart failure	45 (39%)	17 (38%)	10 (20%)	18 (90%)	<.001
Myocardial infarction	31 (27%)	10 (22%)	6 (12%)	15 (75%)	<.001
**Cardiomyopathy**					
Dilated non-ischemic	15 (13%)	7 (15%)	4 (8%)	4 (20%)	.329
Ischemic	29 (25%)	10 (22%)	5 (10%)	14 (70%)	<.001
Hypertrophic	11 (9%)	4 (9%)	6 (12%)	1 (5%)	.654
Valvular	24 (21%)	13 (28%)	10 (20%)	1 (5%)	.090
Congenital	0	0	0	0	-
**Atrial fibrillation**					
Paroxysmal	21 (18%)	9 (20%)	9 (18%)	3 (15%)	.889
Persistent	8 (7%)	1 (2%)	6 (12%)	1 (5%)	.162
Permanent	13 (11%)	8 (17%)	4 (8%)	1 (5%)	.200
**LVEF (%) at admission (± SD)**	54% **±** 15.4%	54% **±** 14.7%	60% **±** 12%	38% **±** 3.8%	<.001

LBBAP = left bundle branch area pacing; RVAP = right ventricular pical pacing; ICD = implantable cardioverter-defibrillator.

**Table 2:** Baseline electrocardiogram

	LBBAP n = 45	RVAP n = 50	ICD n = 20	*P*-value
Sinus dysfunction	6 (13%)	13 (26%)	0	.256
2^nd^ degree AV block	8 (18%)	8 (16%)	0	.137
3^rd^ degree AV block	14 (31%)	11 (22%)	2 (10%)	.170
Slow atrial fibrillation	2 (4.5%)	3 (6%)	0	.538
AV node modulation	4 (9%)	1 (2%)	0	.149
Tachy-brady syndrome	3 (6.5%)	7 (14%)	0	.141
Sinus rhythm	8 (18%)	4 (2%)	17 (85%)	<.001
Atrial fibrillation with normal heart rate	0	3 (6%)	1 (5%)	.258
**Associated bundle branch**				-
LBB 130ms-150ms	9 (20%)	5 (10%)	6 (30%)	.115
LBB > 150ms	6 (13%)	2 (4%)	2 (10%)	.266
Undifferentiated bundle branch > 150ms	1 (2%)	1 (2%)	0	.804
RBB + Left anterior fascicular block	3 (6.5%)	5 (10%)	0	.330

LBBAP = left bundle branch area pacing; RVAP = right ventricular apical pacing; ICD = implantable cardioverter-defibrillator; LBB = left bundle branch; AV = atrioventricular.

Patients who did not have on the baseline ECG an associated bundle branch all benefited of a LBBAP device because their LVEF was at minimum, mildly altered.

The RVAP group includes patients implanted with a right ventricular Apex lead, with or without a probe in the coronary sinus. The ICD group includes patients implanted with an ICD lead, with or without a probe in the coronary sinus.

**Leads impact on the TVA.** Overall, 49 of 115 patients (42.6%) presented with lead–leaflet impingement (Table 3), 9 of 20 patients (45%) in the ICD group, predominantly involving the posterior leaflet (6/20 cases, 30%), 19 of 50 patients (38%) in the RVAP group, also predominantly affecting the posterior leaflet (14/50 cases, 28%), and 21 of 45 patients (46.7%) in the LBBAP group, in which anterior leaflet impingement was more frequently observed (7/45 cases, 15.6%). No anterior leaflet impingement was observed in either the RVAP group or the ICD group.

**Table 3:** Lead’s TV crossing site (see figures below)

	ICD (n = 20)	RVAP (n = 50)	AAL (n = 70)	LBBAP (n = 45)	*P*-value	*P*-value LBBAP vs Apex	*P*-value LBBAP vs AAL
**Anterior crossing site (n)**	2 (10%)	6 (12%)	8 (11.5%)	19 (42%)	*P* < .001	.002	*P* < .001
**Septoposterior crossing site (n)**	15 (75%)	35 (70%)	50 (71.5%)	22 (48%)	.047	.059	.025
**Impingement (n)**	9 (45%)	19 (38%)	28 (40%)	21 (46%)	.676	.518	.608
Posterior leaflet	6 (30%)	14 (28%)	20 (28%)	5 (11%)	.085	.072	.047
Septal leaflet	0	3 (6%)	3 (4.5%)	5 (11%)	.251	.599	.304
Anterior leaflet	0	0	0	7 (15%)	.003	.012	.002
Anteroseptal commissure	1 (5%)	0	1 (1.5%)	0	.091		1.000
Anteroposterior commissure	1 (5%)	1 (2%)	2 (3%)	2 (4%)	.745	.926	1.000
Posteroseptal commissure	1 (5%)	1 (2%)	2 (3%)	1 (2.5%)	.760	1.000	1.000
Interposterior commissure	0	0	0	1 (2.5%)	.456	.958	.823
**No Impingement (n)**	8 (40%)	22 (44%)	30 (43%)	20 (44%)	.941	1.000	1.000
Posteroseptal commissure	7 (35%)	13 (26%)	20 (28%)	7 (15%)	.199	.320	.167
Anteroseptal commissure	0	1 (2%)	1 (1,5%)	0	.519	1.000	1.000
Anteroposterior commissure	0	2 (4%)	2 (3%)	3 (7%)	.471	.904	.610
Interposterior commissure	0	3 (6%)	3 (4,5%)	3 (7%)	.508	1.000	.896
Central	1 (5%)	3 (6%)	4 (6%)	7 (15%)	.214	.238	.154
**Not visualized (n)**	3 (15%)	9 (18%)	12 (17%)	4 (10%)	.435	.321	.331

LBBAP = Left Bundle Branch Area Pacing; RVAP = Right Ventricular Apical Pacing; ICD = Implantable cardioverter-defibrillator; AAL = all apical leads; Anterior crossing site includes anterior leaflet impingement, anteroposterior commissure (with or without impingement) and central positions; Septoposterior crossing sites includes all the other positions (with or without impingement).

Note: One patient received both an ICD lead and a left bundle branch lead; we decided to classify this case in the left bundle branch group.

**Figure 1:**
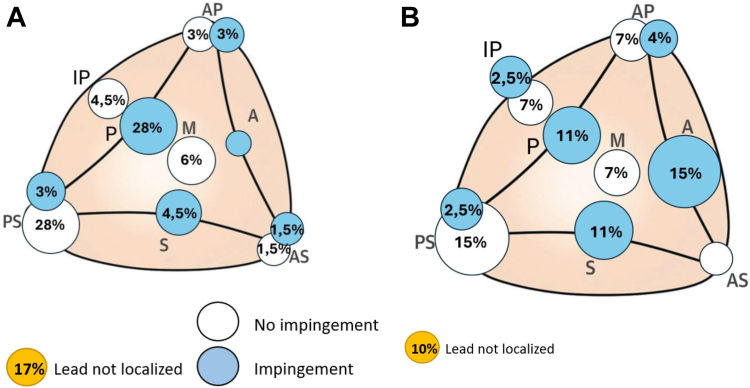
A: Apex lead’s crossing site. **B:** LBB lead’s crossing site.

**Figure 2:**
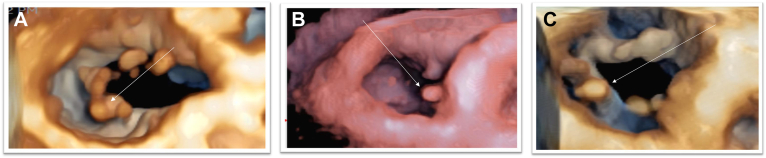
Lead crossing sites on 3D transthoracic echocardiography examples. **A:** Quadricuspid valve, lead in the interposterior commissure. *Arrow:* Lead. **B:** Septal leaflet impingement. *Arrow:* Lead. **C:** Posterior leaflet impingement. *Arrow:* Lead.

Impingement was found in 28 of 70 (40%) patients with apical ICD or PPM leads (all apical leads, all apical leads [AAL]), and 21 of 45 (46%) in the LBBAP group (*P* = .608) (Figure 1A and 1B). For LBBAP, leads crossed the TV more often in the anterior area compared with AAL (*P* < .001), and to apical pacing leads alone (*P* = .002), whereas AAL crossed more often in the postero-septal area (*P* = .025). In 16 of 115 cases (13.9%), the lead was not adequately visualized, 3 of 20 (15%) in the ICD group, 9 of 50 (18%) in RVAP group, and 4 (8.9%) in LBBAP group (*P* = .435) (Figure 2).

**Figure 3:**
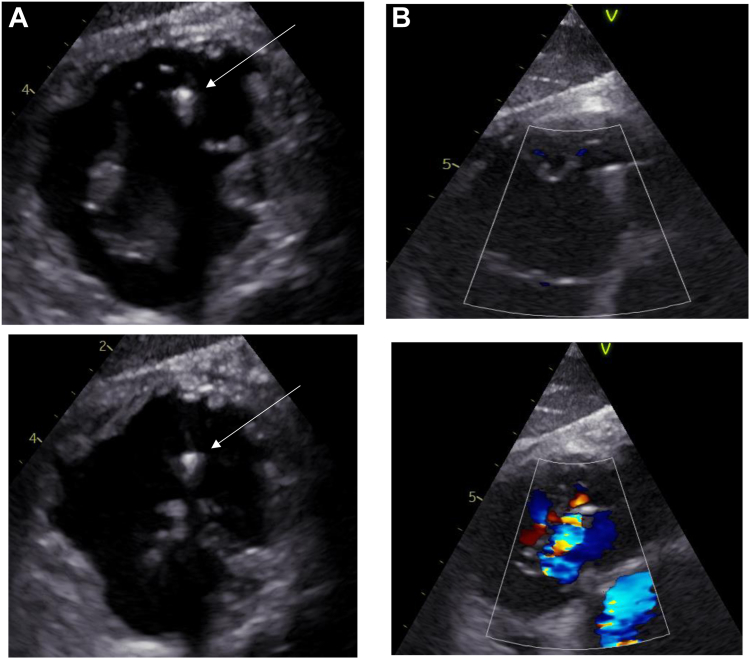
**A:** TEE transgastric view in of a LBB lead through the tricuspid valve, with a conflict in the interposterior commissure. *Arrow:* Lead in the interposterior commissure. **B:** Tricuspid regurgitation related to the LBB lead leaflet impingement seen in transgastric view.

**Leads-related TV regurgitation (**Figures 3 **and** 4**) (**Table 4**).** Among the 49 of 115 (42.6%) patients with leaflet or commissural impingement identified on the immediate post-implantation TTE, 5 of 26 exhibited worsening of TR from grade 1 to significant TR (≥grade 2) at the 6-month follow-up (10.2%, *P* = .016): 2 in the RVAP group and 3 in the LBBAP group. No patient without initial impingement showed worsening TR at follow-up. One patient experienced a post-implantation-related severe TR after a left bundle branch pacing failure, which was subsequently completed with coronary sinus lead implantation. The mechanism appeared to be a subvalvular laceration leading to posterior leaflet prolapse. This patient unfortunately required 2 hospitalizations for heart failure during the follow-up period.

**Figure 4:**
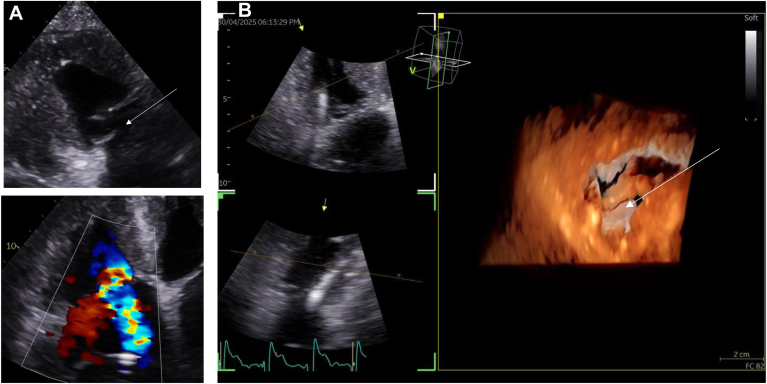
**A:** Flail tricuspid leaflet after leaflet apparatus laceration. *Arrow:* flail tricuspid leaflet. **B:** 3D TTE view of the TV laceration. *Arrow:* flail tricuspid leaflet.

**Table 4:** Worsening or new-onset lead-related TR associated at follow-up

	Total (n = 66)	ICD (n = 14)	RVAP (N = 36)	LBBAP (n = 16)
**NO IMPINGEMENT (n):**	40 (61%)	7 (50%)	21 (59%)	12 (75%)
Worsening of TR	0	0	0	0
No worsening of TR	40 (61%)	7 (50%)	21 (59%)	12 (75%)
**IMPINGEMENT (n):**	26 (39%)	7 (50%)	15 (41%)	4 (25%)
No worsening of TR	21 (31%)	7 (50%)	13 (36%)	1 (6%)
One grade worsening TR	5 (8%)		2 (5%)	3 (19%)
**LEAFLET IMPINGED:**				
Septal	2		1	1
Posterior	2		1	1
Posteroseptal commissure	1			1
**TIMING FOR TR WORSENING**				
1 day after implantation			∗	
At 3–6 months follow up	2		2	3

LBBAP = left bundle branch area pacing; RVAP = right ventricular apical Pacing; ICD = Implantable cardioverter-defibrillator.

Notes: 66 patients underwent follow-up; however, the 6-months evaluation was limited to only 26 patients presenting with leaflet impingement on the post immediate echocardiography.

∗ One case of severe tricuspid regurgitation has been observed one day after implantation. It was attributed to failure in left bundle branch implantation.

**Effect of pacing modality on LV remodeling (**Table 5**).** 71 of 115 patients (62%) were permanent paced on post-implantation TTE: n = 20 with RVAP alone, n = 14 with CRT, and n = 37 with LBBAP. In the fully-paced population (100%), no significant differences were observed between the LBBAP and RVAP or CRT groups in the early post-implantation changes (Δ) in LVEDVi (*P* = .728) or LVESVi (*P* = .102). However, SVi was significantly lower in the RVAP group compared with the LBBAP group (*P* = .034). Comparisons between the LBBAP and CRT groups revealed no significant early differences in ΔLVEDVi (*P* = 1.000), ΔLVESVi (*P* = 1.000), or ΔSVi (*P* = 1.000). Overall analysis demonstrated a significant difference among groups for the post-implantation ΔGLS (*P* < .001). Post hoc testing indicated non-significant worse GLS in the RVAP group compared with both the LBBAP (*P* = .011) and CRT (*P* = .117) groups. Time-to-peak strain dispersion (tPSD) was also significantly different between groups (*P* < .001), with a trend toward higher tPSD in the RV apically paced group compared with the LBBAP group (*P* = .053), and a significantly higher tPSD in the RVAP group compared with the CRT group (*P* = .001). ΔLVEF was –3% in the RVAP paced group vs +1% in the LBBAP group (*P* = .021), while the LBBAP and CRT groups had similar ΔLVEF (*P* = 1.00).

**Table 5:** Early LV volumes and GLS variation after pacing

Variable (min; max) [Q1-Q3]	RVAP (N = 20)	CRT (N = 14)	LBBAP (N = 37)	*P*-value	LLBAP vs RVAP	LBBAP vs CRT	RVAP vs CRT
delta LVEDVi, median	-5 (-29; 19) [-15 to 2.5]	-3 (-20; 8) [-7.75 to 0]	-3 (-30; 14) [-9 to 3]	.728	1.000	1.000	1.000
delta LVESVi, median	1 (-10; 26) [-2.25 to 6.25]	-2 (-12; 16) [-5.5 to -1]	-1 (-20; 20) [-7 to 3]	.102	0.235	1.000	0.155
delta SVi, median	-6 (-23; 11) [-15.25 to -2.75]	-3 (-12; 9) [-7.5 to 2]	-1 (-23; 16) [-5 to 3]	.037	0.034	1.000	0.293
delta GLS, median	6.45 (-4.7; 14.1) [2.07 to 8]	-0.85 (-6.7; 1.7) [-2 to 0.8]	1 (-3.4; 23) [-1 to 3]	<.001	0.011	0.117	<0.001
delta tPSD, median	11.75 (-68; 103.7) [-4.15 to 34.15]	-25.95 (-110; 16) [-42.5 to -13.5]	-5 (-436; 143) [-28 to 11]	<.001	0.053	0.131	0.001
delta LVEF, median	-3 (-20; 10) [-7 to -1.75]	0 (-4; 8) [-2.75 to 4.5]	1 (-21; 13) [-4 to 4]	.011	0.021	1.000	0.037

LBBAP = left bundle branch area pacing; RVAP = right ventricular apical pacing; ICD = implantable cardioverter-defibrillator; CRT = cardiac resynchronization therapy; LVEDVi = indexed left ventricular end-diastolic volume; LVESVi = indexed left ventricular end-systolic volume; SVi = indexed stroke volume; GLS = global longitudinal strain; tPSD = time-to-peak strain dispersion; LVEF = left ventricular ejection fraction.

**Figure 5:**
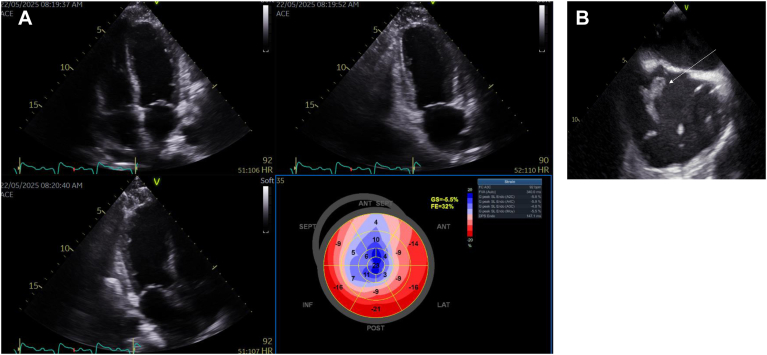
**A:** Tako Tsubo 1 day after implantation (3 cases). **B:** Thrombi 1 day after implantation in TEE (1 case). *Arrow:* Thrombi.

**Complications.** Post-implantation TTE also revealed several rare complications. A CIED-related Takotsubo syndrome was unexpectedly identified in 3 patients (Figure 4): in 2, no apparent cause was found, and in the third, it occurred following a post-implantation pericardial effusion requiring pericardial drainage observed in the RVAP. At the 8-week follow-up, TTE demonstrated complete recovery of LV systolic function, thereby confirming the diagnosis in all 3 cases. In addition, a large right atrial thrombus was discovered on post-implantation TTE and subsequently confirmed by TEE (Figure 5). The thrombus was unexpectedly attached to the interatrial septum, near a large patent foramen ovale. Computed tomography revealed bilateral pulmonary emboli but no thrombus along the venous pathway of the leads. Laboratory testing showed no evidence of thrombophilia. No clear cause of this thrombus was identified, and treatment with a direct oral anticoagulant was initiated. Complete thrombus resolution was documented 4 weeks later.

## Discussion

### Major findings

This study prospectively evaluate the impact of LBBAP vs RVAP on TVA lead-crossing localization. Our results showed that LBBAP leads more frequently crossed the TV at an anterior site, whereas RVAP leads tended to cross in a more postero-septal position. The early incidence of new-onset LITR or WTR appeared to be low (4.3%, 5/115) but represented 10.2% (5/49) of patients with leaflet or commissural impingement identified on immediate post-implantation TTE. An immediate beneficial effect of LBBAP on indexed LV stroke volume (SVi), post-implantation global longitudinal strain (ΔGLS), and time-to-peak strain dispersion was also evidenced. Finally, early systematic TTE to detect complications is essential after device implantation particularly LBBAP.

**Pacing mode leads influence on the TVA.** It remains unclear whether lead location influences the development of TR. Some reports have suggested a lower incidence of TR with RV outflow tract pacing compared with apical pacing.^1^^,^^13^, ^14^, ^15^, ^16^ Echocardiographic and post-mortem studies have demonstrated that device leads may disrupt the TVA by impinging on or adhering to a leaflet, interfering with the sub-valvular apparatus, or causing leaflet perforation, laceration, or avulsion, the latter occasionally occurring during lead extraction.^1^^,^^13^, ^14^, ^15^, ^16^ Post-mortem and surgical observations further show that fibrosis can entrap the lead within the leaflet and/or sub-valvular apparatus, leading to leaflet mal-alignment and mal-coaptation. Although not a direct form of lead-induced TV dysfunction, the presence of a lead may also predispose to thrombus formation and endocarditis.^1^^,^^13^, ^14^, ^15^, ^16^ They are conflicting data as to whether patient- related clinical and echocardiographic data can predict lead-related TR. Accordingly, our study represents a new investigation in order to determine whether there is device lead-mediated interference with the TV leaflets, especially based on the pacing mode and lead position. In order to improve our analysis, we used a combined 2D and 3D-TTE dataset. Thus, we observed that subxiphoid 2D-TTE acquisitions were useful for locating the lead, particularly in the LBBAP group, as the very thin leads were often difficult to visualize with 3D-TTE in apical and parasternal views.^15^^,^^16^ These observations may lead us to rely increasingly on sub xiphoid 2D-TTE for improving the lead localization. The systematic use of 3D-TTE was initially planned to provide the most accurate visualization possible of both the lead and TV anatomy, as reported in earlier studies.^8^ Previous studies have shown that 3D-TTE would more effective and better accepted than TEE for tracking the lead as it crosses the TV, which is why we mainly limited our imaging protocol to this modality.^8^^,^^15^^,^^16^ What is frequently described is that the posterior and anterior TV leaflets are visualized in the RV-inflow view, whereas the septal and anterior leaflets are seen in both the parasternal short-axis and the apical 4- chamber views.^1^^,^^15^^,^^16^ The anatomy variability may also explain why similar CIED lead positions and types can lead to worsening TR in some patients but not in others.^15^ The TV coaptation length averages 5–10 mm, providing a “coaptation reserve.” This may explain why, in a healthy heart with mild lead–leaflet interaction, valve coaptation remains preserved and TR does not worsen immediately, but may develop later as the tricuspid annulus, RV, and right atrium (RA) progressively dilate.^1^, ^2^, ^3^ 3-dimensional echocardiography allows precise visualization of CIED leads relative to the TV. Leads may be positioned in commissures, against or adherent to a leaflet, or across the central valve. Impinging or adherent leads are recognized to be strongly associated with significant TR, whereas commissural or central positions are less often implicated.^1^, ^2^, ^3^^,^^12^^,^^13^ Impinging leads directly disrupt leaflet coaptation, while adherent leads remain fixed to the apparatus but move with it. Multivariable analyses have identified pre-implantation vena contracta width and interfering leads as independent predictors of post-implantation TR.^4^ CIED-induced TR should be suspected in any patient with a device lead and new significant TR. Diagnostic clues on 3D-TTE include a regurgitant jet hugging the lead, leaflet mal-coaptation, adherence to subvalvular structures, marked septal displacement, or non-RV outflow tract positioning. In our prospective analysis, the anatomical trajectory of LBBAP and RVAP naturally leads to different sites of tricuspid leaflet interaction. However, to our knowledge, our study is the first to provide quantitative echocardiographic evidence supporting this anatomical rationale in a clinical cohort. By documenting the frequency and specific leaflet involvement (42% anterior for LBBAP vs 71.5% posteroseptal for RVAP), we provide data that could inform future lead placement strategies and improve understanding of pacing-induced TR mechanisms. Although our study does not propose new clinical protocols, it establishes an evidence-based foundation for such developments in subsequent research.

**Impact of LBBAP on the TR risk**. Huang et al^17^ are considered the conceptual pioneers of the transseptal approach for LBBAP, which overcame many deleterious effects of RVAP.^17^, ^18^ The term LBBAP has been adopted because deep septal pacing can generate variable ventricular activation patterns, and confirming that perfect left bundle branch physiological conduction system capture remains challenging.^17^, ^18^ Conduction system pacing (CSP) provides a more physiological mode of ventricular activation and has been associated with superior echocardiographic parameters and improved clinical outcomes compared with conventional RVAP. Several studies have even suggested that CSP may be more effective than CRT.^19^^,^^20^ Compared with standard apical pacing, LBBAP has been consistently associated with higher post-implantation LVEF, lower dyssynchrony index, reduced myocardial work, and less impairment in GLS.^19^^,^^20^ Similarly, when compared with CRT, LBBAP has demonstrated superior LVEF and greater functional improvement.^17^, ^19^, ^20^ A recent meta-analysis comparing LBBAP with CRT confirmed these findings, showing improved echocardiographic features, enhanced functional status, a lower rate of hospitalization for heart failure (HHF), and better survival.^20^ The main mechanism proposed to explain this superiority is improved LV synchrony achieved with LBBAP.^19^^,^^20^ In this clinical context, TR after CIED implantation remains a concern, likely because of lead impingement or underlying cardiac abnormalities. The prevalence of new-onset or worsening TR ranges from 20% to 50% compared with patients without a pacemaker.^1^^,^^3^^,^^5^, ^6^, ^7^, ^8^ Tricuspid valve dysfunction after conventional pacemaker therapy is generally attributed to mechanical interaction of the transvalvular lead with the TVA, traumatic injury during implantation, or cardiac remodeling secondary to heart failure.^1^^,^^3^^,^^5^, ^6^, ^7^, ^8^ To date, only a few studies have investigated interactions between LBBAP leads and the TVA.^12^, ^13^, ^14^^,^^21^ Regarding pacing modalities, in a retrospective study,^15^ non-apical RV pacing, mainly mid septal pacing, was associated with less worsening TR and a crossing more frequent at the middle of the TV, whereas apical pacing was associated with more posterior crossing.^15^ Nevertheless, the high rate of severe CIED-related TR was much higher than usually reported in the literature, making it poorly generalizable. In this study, a higher lead TV crossing assessed by X-Ray during the implantation was a risk factor of worsening CIED-related TR.^15^ Li et al^13^ reported a similar risk of TR progression between LBBAP and RVAP; in their study, the electrode-to-tricuspid (E-T) distance was the main predictor of TR worsening in LBBAP. The risk of LBBAP-related TR progression increased when the lead was positioned too close to the tricuspid annulus.^13^ The authors observed post-procedure moderate TR because of impingement of the septal leaflet by the LBBAP lead with an E-T distance of 11 mm, whereas a distance >19 mm was associated with a lower risk of TR progression.^13^ A second study, including 89 patients implanted with an LBBAP device, reported a 32% incidence of TR worsening by at least one grade.^21^ A septal fixation distance of <16 mm from the tricuspid annulus was also identified as an independent risk factor for post-implantation TR progression.^21^ Using 2D/3D-TTE, the authors also found that lead crossing in the mid-valve or septoposterior position was protective against new or worsening TR. Lead–leaflet interference was observed in 45% of patients and emerged as an independent risk factor for TR progression, with a stronger impact than tricuspid annular (TA) diameter and right atrial (RA) dimensions, increasing the risk of moderate-to-severe TR by 11- to 15-fold.^21^ The septal leaflet was most frequently affected (51%). Our findings suggest that early mechanical interference of LBBAP leads with the subvalvular apparatus does not influence short-term TR risk. The role of RVAP using conventional transvenous leads in TR pathogenesis also remains controversial. Multiple additional factors likely contribute to long-term secondary TR, including pacing-induced dyssynchrony leading to LV diastolic and systolic dysfunction, or mitral regurgitation, which increases left-sided filling pressures and pulmonary artery pressures, ultimately resulting in functional TR. Finally, the immediate risk of severe TR after CIED implantation, including LBBAP, appears to be low after having eliminated in the inclusion criteria those who already had a TR. However, robust longitudinal studies are needed to clarify the long-term impact of pacing modality—particularly LBBAP—on TR progression.^22^ Accordingly, the routine post-implantation TTE should be considered to detect the lead impinging location. Thus, a new algorithm was proposed last year, advocating systematic echocardiographic assessment both before and shortly after implantation.^23^^,^^24^ This approach assumes that routine post-implantation TTE should be considered to detect early severe CIED-related TR and discuss early lead repositioning or TLE when appropriate, to prevent rare but potentially serious consequences.^25^^,^^26^ In our study, only 1 patient developed early worsening TR, which was massive and clinically significant. Unfortunately, despite early detection, no effective therapeutic intervention could be undertaken in our elderly and frail patients. However, if the patient had been younger and surgically eligible, prompt intervention could potentially have changed the prognosis. Furthermore, the 3 cases of Takotsubo syndrome, the pericardial effusion, and the large right atrial thrombus observed in our cohort raise questions regarding the optimal early post-implantation management strategy. Although the incidence of such complications was low, their clinical relevance was considerable.^3^^,^^25^, ^26^, ^27^ Early detection could have allowed us to prevent more severe later complications. For instance, the patient with the right atrial thrombus had no prior indication for oral anticoagulation (OAC); early diagnosis and anticoagulation allowed prompt management.

**Early benefits effect of LBBAP.** The physiological advantages of LBBAP are driving worldwide adoption, alongside emerging guidance on indications and implant techniques. Based on observational evidence, LBBAP has been incorporated into clinical guidance. The 2023 Heart Rhythm Society/Asia Pacific Heart Rhythm Society/Latin American Heart Rhythm Society consensus gives a class IIa recommendation for CSP for bradycardia indications in patients with a LVEF between 35% and 50% who are expected to require substantial ventricular pacing.^28^ Early observational studies of LBBAP-CRT reported QRS narrowing and improvements in left ventricular (LV) function and volumes, particularly in non-ischemic cardiomyopathy.^28^, ^29^, ^30^, ^31^ In a recent multicenter observational study of 325 patients undergoing CRT, LBBAP—compared with biventricular pacing (BVP-CRT)—was associated with greater LVEF improvement and shorter QRS duration, and with better survival, despite baseline differences between groups.^31^ Moreover, in this field, to our knowledge, no data specifically addressing immediate remodeling end points are currently available. The few existing studies have primarily evaluated early LV remodeling within 3–6 months after implantation. In one prospective cohort of patients undergoing LBBAP, echocardiographic assessment demonstrated a significant improvement in LVEF, from 52.9 ± 10.6% at baseline to 56.9 ± 8.4% at 3 months (*P* = .004), accompanied by a reduction in time-to-peak strain dispersion (tPSD), indicating an early favorable electromechanical effect and resynchronization.^31^ Our observations are consistent with these findings but are particularly striking, as we detected very early differences between RVAP and LBBAP in indexed stroke volume (SVi; *P* = .034), tPSD (*P* < .001), and ΔLVEF (–3% in the RVAP group vs +1% in the LBBAP group; *P* = .021), all in favor of LBBAP. Taken together, these results support the presence of very early LV reverse-remodeling signals with LBBAP. Longer follow-up, particularly at 1 year, will help distinguish the immediate electromechanical effects from long-term LV remodeling in terms of morphological parameters.

**Clinical implications.** This study aligns with previous research on lead-related TR mechanisms, confirming the association between lead–leaflet impingement and TR progression. Moreover, we found that LBBAP leads crossed more frequently in the anterior tricuspid area, while RVAP leads crossed more often in the posteroseptal region. In our follow-up population, the incidence of new-onset LITR or WTR was low, but occurred in 19% of patients with leaflet or commissural impingement identified on immediate post-implantation 3D-TTE. This subgroup may be at higher long-term risk for TR. Additionally, identifying very early signs of left ventricular (LV) reverse-remodeling after LBBAP may help predict hemodynamic responders. Finally, our postoperative findings support the use of systematic post-pacing echocardiographic monitoring to detect early and potentially serious complications.^25^, ^26^, ^27^^,^^30^^,^^31^

## Limitations

First, the limited follow-up rate and sample size reduced the statistical power to confirm the association between lead–tricuspid impingement localization based on the pacing mode and the long-term TR risk. Second, localizing the lead crossing the TV during LBBAP is highly challenging, as in some apical CIED implantations, even with advanced 3D-TTE imaging. However, in our study all examinations were performed under optimal laboratory conditions, and patients with poor echogenicity were excluded. Third, because of the relatively small sample size, statistical power may have been limited; nonetheless, this study required substantial workload, particularly for detailed 3D analysis. Fourth, in a small proportion of patients—especially those with LBBAP—the TV crossing site may have been misclassified because of the thin diameter of the leads used. Fifth, although ICD leads are thicker in diameter, several factors may explain the absence of TR worsening in this subgroup despite a similar impingement rate: ICD leads are typically less mobile because of higher stiffness, which may limit repetitive leaflet interference during the cardiac cycle; patients with ICDs generally had different baseline indications and generally RV dilatation, the small difference in the diameter leads size is counterbalanced by the cavity dilatation. Sixth, the very small number of events precluded an adjusted multivariable regression analysis. Although this limits the ability to control for potential confounders, the consistent direction of the association suggests a potential effect that warrants confirmation in larger cohorts. Finally, this study did not include the distance between the pacing lead and the tricuspid annulus, a parameter previously shown to influence TR risk.^13^^,^^21^ This limitation may partly have influenced the non-significant difference observed and indicates overlap with prior findings. Nevertheless, our study still contributes by quantifying leaflet-specific interactions for LBBAP and RVAP, providing additional echocardiographic context that complements existing research and can serve as a foundation for more comprehensive future studies including lead–annulus distance as a variable.

## Conclusion

This prospective study found that LBBAP leads cross the TV more frequently in the anterior area, whereas RVAP leads tend to cross in a posteroseptal position. Early worsening of TR after CIED implantation was uncommon but could be anticipated through early detection of leaflet or commissural impingement on 3D-TTE. The relationship between the initial LBBAP lead position on the TVA and long-term TR remains to be determined. Our findings support the systematic use of post-implantation echocardiographic follow-up to detect early complications and better characterize lead–TVA interactions.

## Disclosures

The authors have no conflicts of interest to disclose.
